# Binding of the *Helicobacter pylori* OipA causes apoptosis of host cells via modulation of Bax/Bcl-2 levels

**DOI:** 10.1038/s41598-017-08176-7

**Published:** 2017-08-14

**Authors:** Omid Teymournejad, Ashraf Mohabati Mobarez, Zuhair Mohammad Hassan, Amin Talebi Bezmin abadi

**Affiliations:** 1 Department of Bacteriology, Faculty of Medical Sciences, Tarbiat Modares University, Tehran, Iran; 2 Department of Immunology, Faculty of Medical Sciences, Tarbiat Modares University, Tehran, Iran

## Abstract

The *H*. *pylori* outer inflammatory protein A (OipA) is an outer membrane protein that contributes to gastric inflammation. OipA is believed to affect intra-cellular signalling and modulate the host signalling pathways. The aim of the current study was to clarify the role of OipA in *H*. *pylori* pathogenesis and its effect on host cell signalling pathways. To this end, the *oipA* gene was isolated and inserted into cloning and expression vectors. The recombinant plasmid was transferred into an expression host to produce OipA, which was subsequently purified by affinity chromatography and used for antibody production. A confluent monolayer of gastric cell lines was treated with various concentrations of OipA and investigated for attachment, toxicity, and apoptosis and alterations in signalling pathways. OipA bound to gastric cell lines confirming its role in the attachment of *H*. *pylori* to host cells. The ratio of Bax/Bcl-2 and caspase3, 8, FasL in the host cells were assessed and the results showed that the Bax/Bcl-2 ratio as well as the level of cleaved-caspase 3 was elevated in OipA-treated cells. These findings suggest that OipA can bind and induce toxic events as well as triggering apoptotic cascade in host gastric cells through intrinsic pathway.

## Introduction

*Helicobacter pylori* is classified as a class I carcinogen by the World Health Organization (WHO) and International Agency for Research on Cancer (IARC)^1^. This bacterium is associated with diseases such as chronic gastritis, peptic ulcer, gastric adenocarcinoma and mucosa-associated lymphoid tissue (MALT). Despite the high prevalence of infections worldwide, the majority of *H*. *pylori* carriers will stay asymptomatic during their lifetime^2^. Although *H*. *pylori* was discovered more than 30 years ago, the basic aspects of its pathogenesis still remain undefined^3^. Prognosis of a *H*. *pylori*–associated disease is believed to be influenced by bacterial virulence factors, host genetic and environmental factors^4^. Several specific *H*. *pylori* virulence factors have been identified^5^. Proteins such as CagA, VacA, and OipA have been associated with more severe gastroduodenal diseases. Furthermore, there are numerous reports in the literature on virulence factors modulating intracellular signalling pathways^6^ or triggering apoptosis in host cells^7, 8^.

The outer inflammatory protein A (OipA) is believed to be one of the major *H*. *pylori* virulence factors; however, status of our knowledge regarding the effects of this protein on the host cells is barely scant. Epidemiological studies have shown that the presence of OipA is associated with duodenal ulcer and gastric cancer. Meanwhile, host-bacteria interaction studies have revealed that this protein induces pro-inflammatory signalling and IL-8 secretion in gastric epithelial cells. The protein also causes neutrophil infiltration, activation of focal adhesion kinase, re-organization of cytoskeleton and dendritic cells suppression^9–11^.

The current study primarily aims to clarify the role of OipA in *H*. *pylori* pathogenesis and to elucidate some of the obscure aspects of cell signalling pathways modulation by this protein.

## Results

### OipA protein

Recombinant OipA was purified by affinity chromatography after induction of *E*.*coli* BL21 containing *oipA* gene by IPTG (Fig. S1). For blocking LPS function in purified OipA solution, polymyxin B sulfate was added to the protein solution and the level of LPS was measured by Limulus amebocyte lysate assay kit. Endotoxin activity was less than 0.25 EU/mL.

Based on our *in-silico* prediction study on OipA, there is a high possibility that OipA (an auto-transporter protein) is inserted and located in outer membrane by type V secretion system (T5SS)^12^. In auto-transporter proteins, beta-barrel regions make a pore in outer membrane and these pores let the N-terminal hydrophilic part pass through the pore. The N-terminal part could either be cleaved or stay bound to the beta-barrel region of protein^12^. Although we don’t know whether OipA N-terminal hydrophilic part is secreted or bound to the beta-barrel regions, we believe that in terms of pathogenesis and binding to host cell receptors, the N-terminal hydrophilic part is the most important part of OipA (Fig. S2). For having the most similar structure with native OipA, we set the following designs; 1- we designed primers right after signal sequence; 2- we did not put His-tag on N-terminal part; 3- we used *NcoΙ* to cut out all extra amino acids which the vector normally adds to the N-terminal part to avoid misfolding of this important part of the protein.

### Rabbit polyclonal antibody titration

Presence of antibody against OipA was measured by enzyme-linked immunosorbent assays (ELISA) test from blood samples obtained on days 0, 35, and 58.ELISA confirmed that antibody titers against OipA increased 58 days after rabbit immunization (Fig. S3).

### OipA binding to gastric cell lines

**Figure 1:**
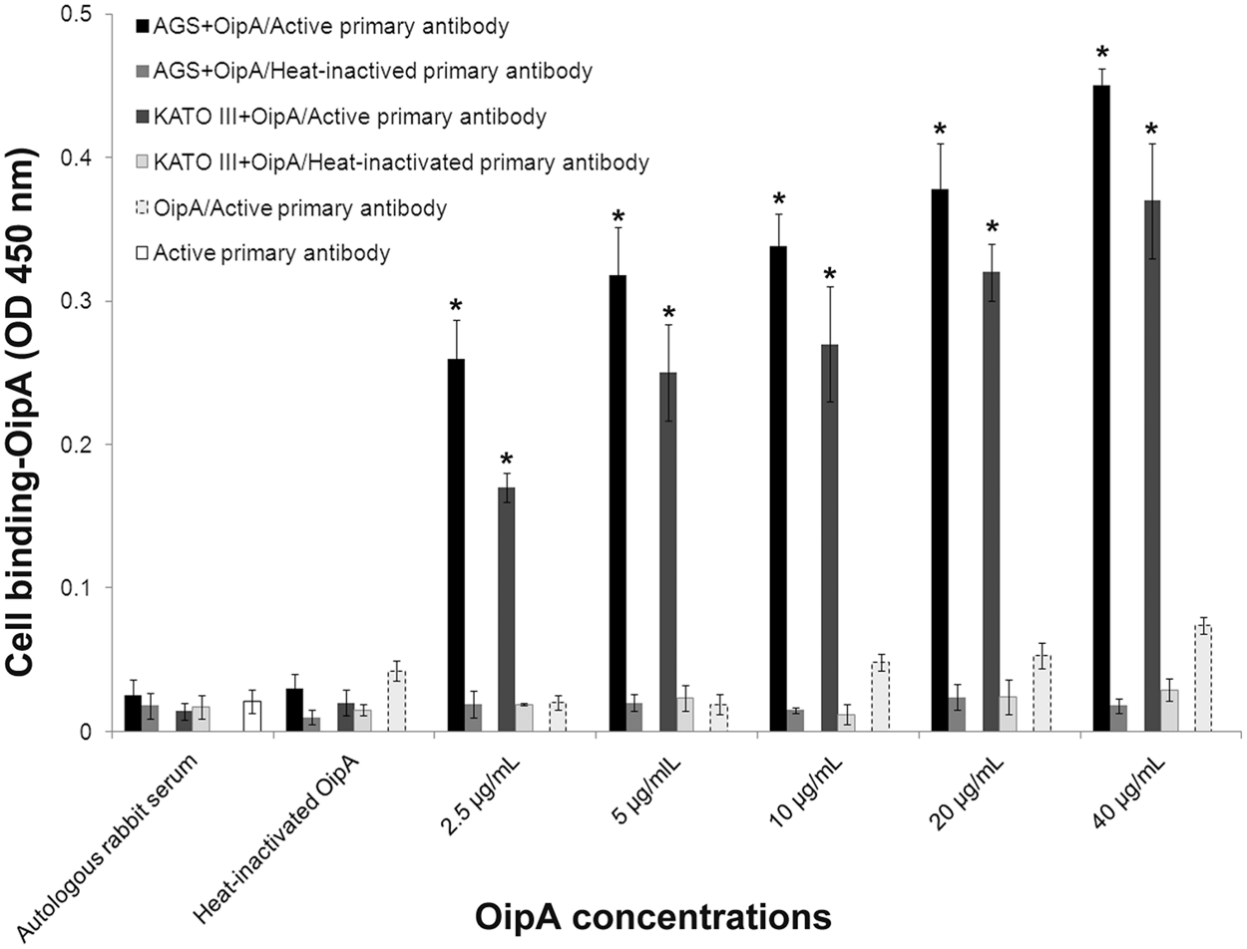
Attachment of various concentrations of OipA protein to AGS and KATO III. ELISA test was performed with rabbit active or heat-inactivated primary antibodies after treatment of AGS and KATO III with various concentrations of OipA or heat-inactivated OipA or autologous rabbit serum as negative controls for 1 h. The results are presented as the mean ± SD, (n = 3, triplicate samples). Statistically significant differences with the control group are indicated with *(p < 0.05).

Various concentrations of OipA were added to AGS and KATO III and the results were compared with the negative controls. Attachment of OipA to gastric cell lines increased with increasing of OipA concentration (Fig. 1). Protein binding was significantly higher for cells treated with 2.5 µg/mL of OipA compared to the negative controls. Furthermore, binding of OipA to AGS cells was more than KATO III (*p* < 0.05).

### OipA is toxic for AGS and KATO III

**Figure 2:**
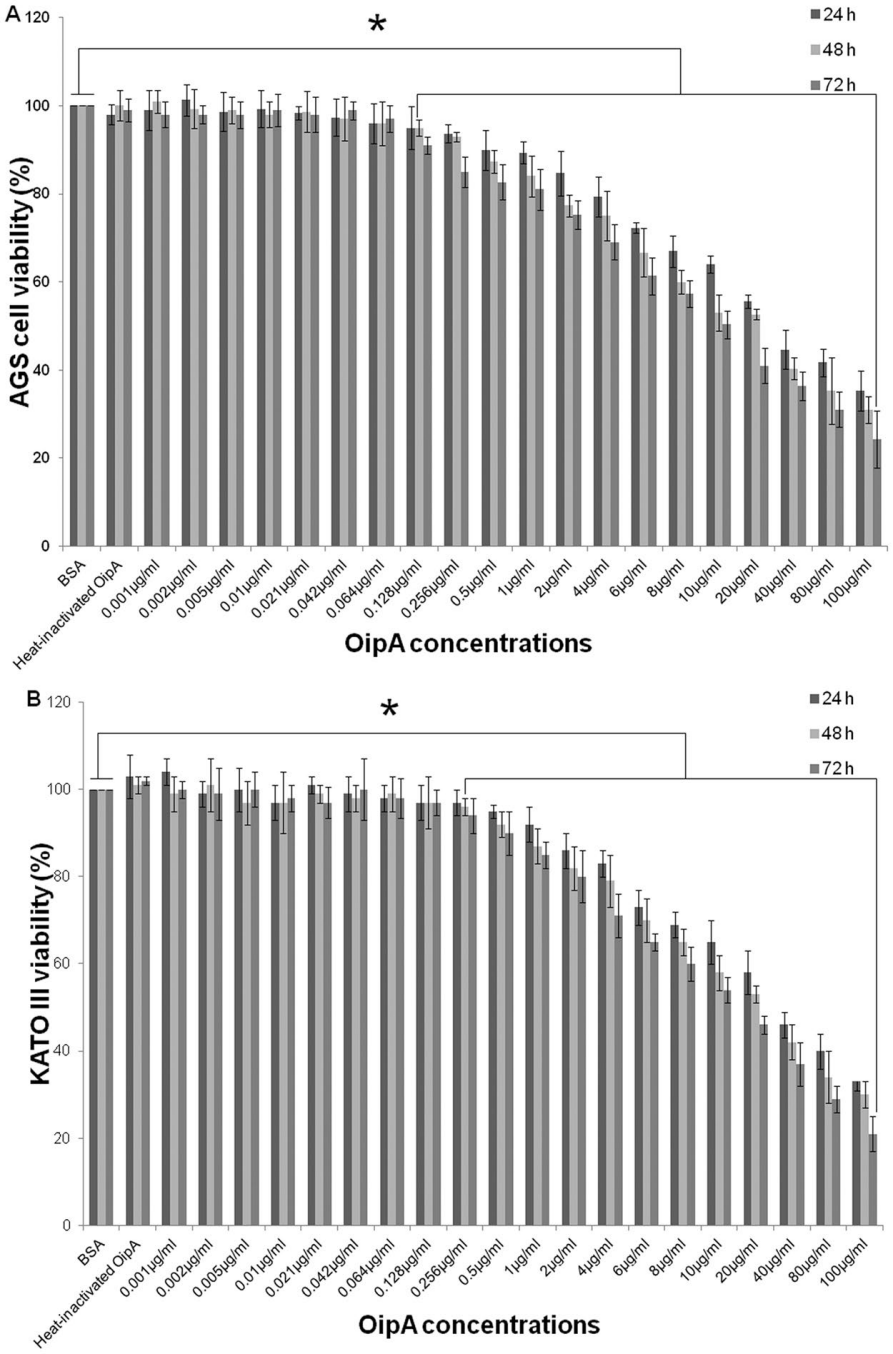
Dose and time dependent toxicity of OipA towards AGS and KATO III. Viability (%) of AGS (**A**) and KATO III (**B**) were measured after 24, 48 and 72 h treatment with various concentrations of *H*. *pylori* OipA or heat-inactivated OipA or BSA as negative controls. The results are presented as the mean ± SD, (n = 3, triplicate samples). Statistically significant differences with the control group are indicated with *(p < 0.05).

Viability of AGS and KATO III was measured with MTT assay after 24, 48, and 72 hrs following treatment of the cells with OipA. The results showed that concentrations of 256 and 500 ng/mL of OipA resulted in significant decrease in viability of AGS and KATO III after 24 hrs of incubation. Lower concentrations of OipA (128 and 256 ng/mL) caused a significant decrease in the viability of AGS and KATO-III when co-incubated for 48 and 72 h. The results indicated that toxicity of OipA on AGS and KATO III is dose and time dependent and OipA has more toxicity on AGS comparing to KATO III (Fig. 2).

### OipA induces apoptosis in gastric cell lines

**Figure 3:**
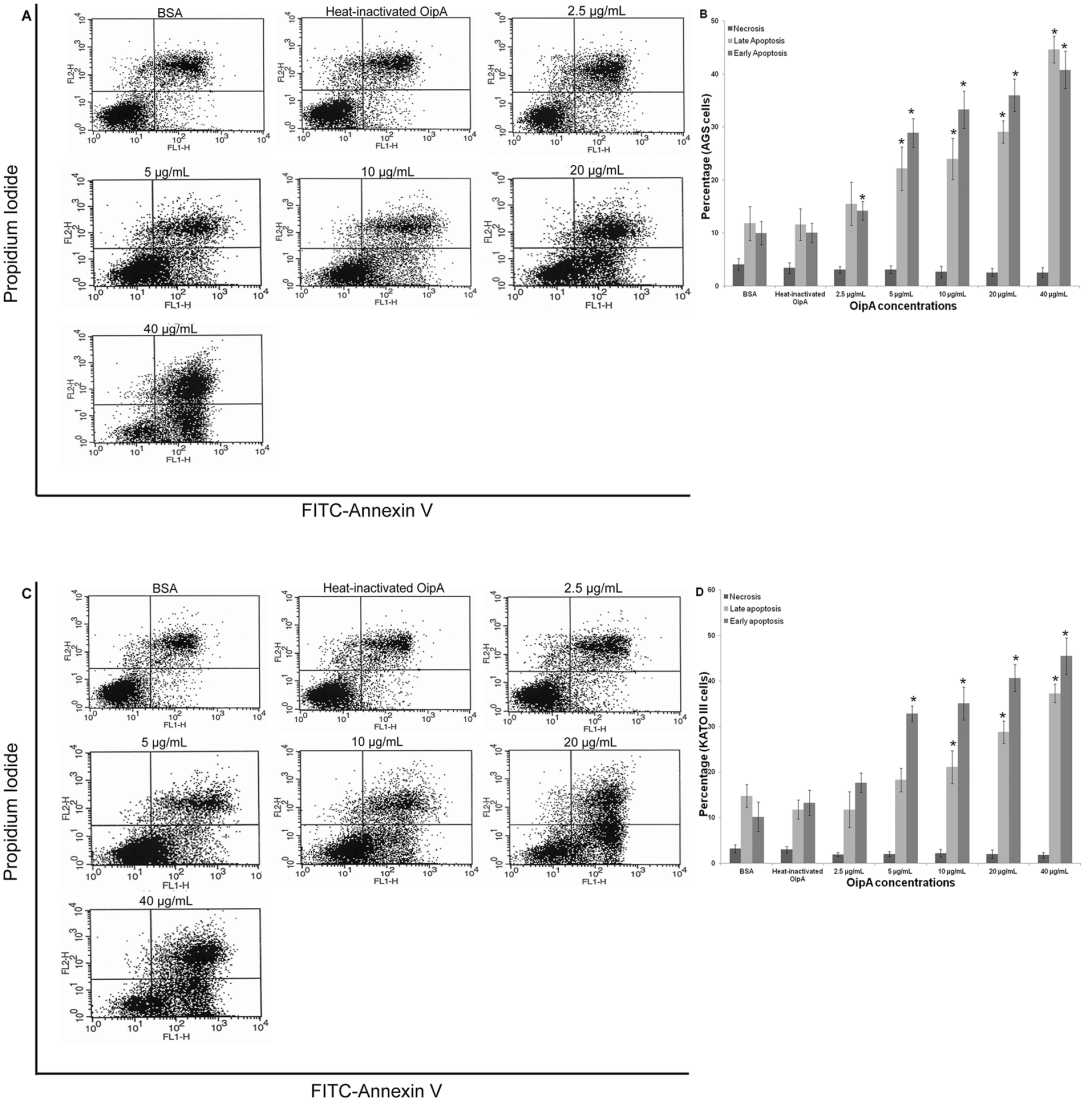
Apoptotic effects (%) of various concentrations of OipA on AGS and KATO III. AGS and KATO III were treated with different concentrations of OipA or heat-inactivated OipA or BSA as negative controls for 24 h and apoptosis was detected by FITC-Annexin V/Propidium Iodide staining. Representative flow-cytometrydot-plots showing the level of apoptosis in AGS (**A**) and KATO III (**C**). Bottom left: live cells, top left: necrosis, bottom right: early apoptosis, top right: late apoptosis. The results are expressed as the mean ± SD, (n = 3, three independent experiments) (**B** and **D**). Statistically significant differences with the control group are indicated with * (p < 0.05).

To investigate apoptosis induction by OipA protein, the protein-treated cells were probed with FITC-Annexin V/PI. FSC versus SSC plots were used for gating cells and for detection of changes in the scatter properties of the cells (Fig. 3A,C). FITC-Annexin V versus Propidium Iodide plots from the gated cells showed populations of viable and non-apoptotic (Annexin V−PI−), early (Annexin V+ PI−), and late (Annexin V+ PI+ ) apoptotic cells (Fig. 3B,D).

OipA concentrations, as low as 5 µg/mL, produced significant difference (*p* < 0.05) with the negative controls in both AGS and KATO III. This effect markedly increased with increasing OipA concentrations.

### OipA induces apoptosis through Bcl-2 family pathway

**Figure 4:**
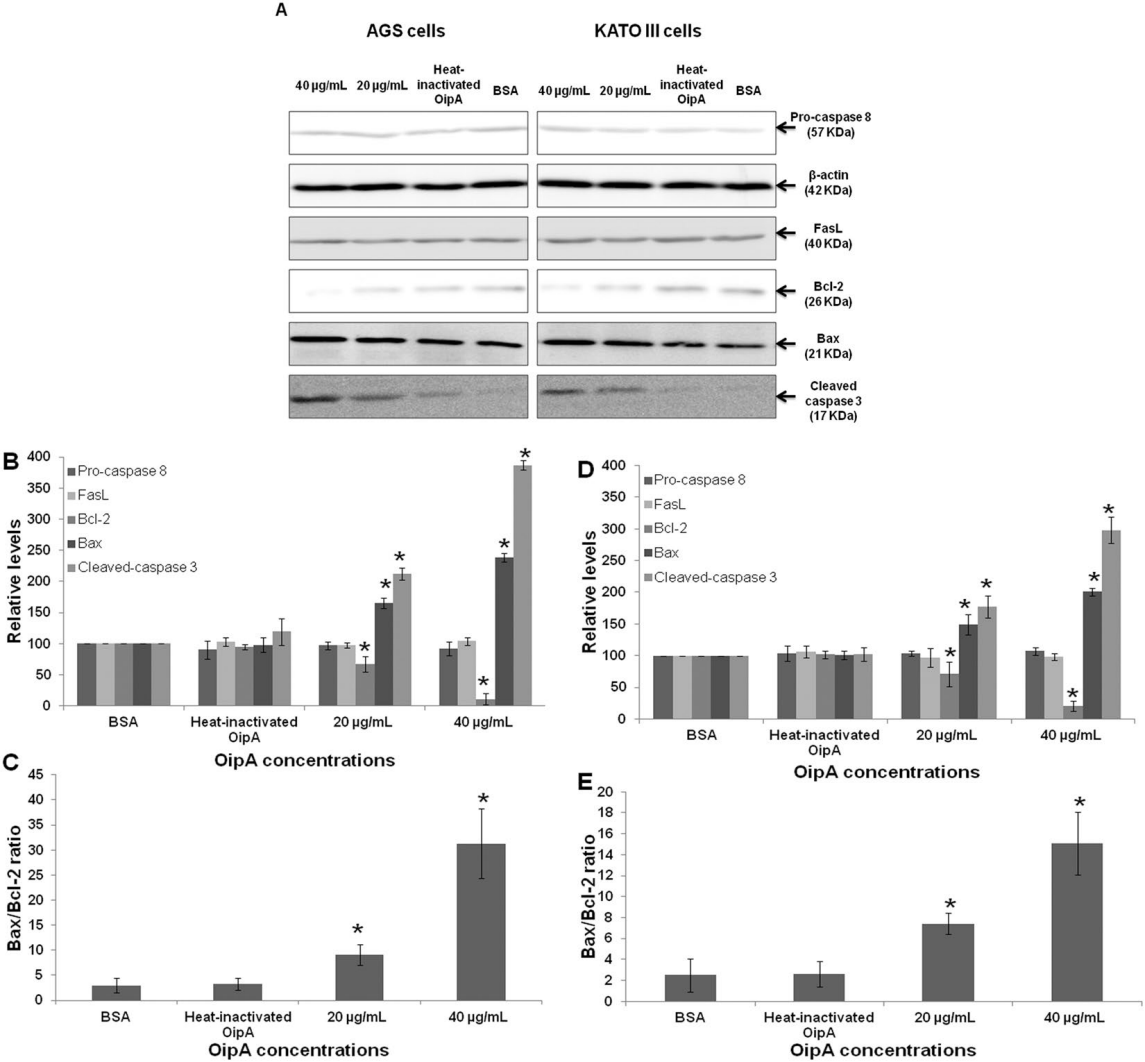
OipA increases cleaved-caspase 3 and Bax/Bcl-2 ratio in AGS and KATO III, but does not have any effect on caspase 8 and FasL. Western blot analysis was performed for determination of caspase 3, 8, FasL and Bax/Bcl-2 ratio in OipA treated AGS and KATO III. Samples were treated with BSA or heat-inactivated OipA as negative controls or 20 μg/mL or 40 μg/ml of OipA for 24 h. Representative pictures showing the level of cleaved-caspase 3, pro-caspase 8, FasL, β-actin, Bcl-2 and Bax (**A**). Densitometric analysis showing the levels of caspase 3, 8, FasL, Bcl-2, Bax in AGS (**B**) and KATO III (**D**) and Bax/Bcl-2 ratio in AGS (**C**) and KATO III (**E**) in all treatment groups. The results are expressed as the mean ± SD, (n = 3, three independent experiments). Statistically significant differences with the control groups are indicated with *(p < 0.05).

Immune blotting revealed that OipA protein induced apoptosis via Bcl-2 family pathway (Fig. 4A). Apoptosis increased with increasing concentrations of OipA. AGS and KATO-III were treated with various concentrations of OipA in 6-well plates for 24 hrs and the level of cleaved-caspase 3, pro-caspase 8, FasL, Bax and Bcl-2 were evaluated by semi-quantitative detection. Quantitative analysis of cleaved-caspase 3, pro-caspase 8, FasL, Bax and Bcl-2 proteins level was performed using a scanning densitometer. The results showed that with an increase of the OipA concentrations, the level of intracellular Bcl-2 declined, and the level of intracellular cleaved-caspase 3 and Bax increased in comparison with the negative controls. Increasing of OipA concentrations did not have any effect on cleavage of pro-caspase 8 and FasL up-regulation (Fig. 4B,D).

## Discussion

The pathogenesis of *H*. *pylori* is associated with modulation of host intracellular signalling pathways^13^. *H*. *pylori* virulence factors such as CagA are believed to interfere with intracellular signalling cascades and is likely the main factor in promoting up-regulation of cell proliferation^14, 15^. CagA and VacA are the best characterized virulence factors in *H*. *pylori*and are involved in gastric inflammation. VacA is a pore-forming toxin and induces apoptosis in specific host cells. This toxin is secreted by type V auto-transporter secretion system and internalized to host cells through endocytosis^16^. *H*. *plori* also has several adhesins for binding to mucosal epithelial cells. Adherence is the first step of colonization and pathogenesis. Moreover, *H*. *pylori* adhesins are considered as bacterial virulence factors. The most well-known *H*. *pylori* adhesions are BabA, SabA, AlpA/B, HopZ and OipA. In this work we investigated the pathogenesis of OipA^16^.

OipA or HopH or OMP13 is an outer membrane protein that is known to induce inflammation and cause IL-8 secretion in host cells^17^. There are some reports suggesting a synergistic effect between OipA and CagA in terms of the pathogenesis^18^.

Our results indicate that OipA can induce apoptosis in gastric epithelial cell lines. Apoptosis is a natural phenomenon that occurs as part of a normal cell life cycle; however, pathogenic bacteria can interfere with these intracellular signalling pathways^19, 20^.Various bacterial proteins, such as VacA, can also induce apoptotic pathways in host cells^21, 22^. In normal cells, there is a balance between apoptosis and proliferation, but in gastric disorders caused by *H*. *pylori*, this balance is altered^23, 24^. This is of prime importance because the imbalance between apoptosis and cell proliferation determines the pathogenesis pathway of *H*. *pylori*; gastric cell apoptosis causes gastric and duodenal ulcers and increasing of gastric cell proliferation leads to metaplasia and cancer^25, 26^.

The Bax/Bcl-2 ratio plays a key role in maintaining the balance between apoptosis and proliferation. Bcl-2 family proteins are an intracellular protein group which affect programmed cell death. Intrinsic or mitochondrial apoptotic cascade follows the Bcl-2 pathway^27^. Our results showed that OipA protein induces apoptosis in AGS and KATO III cells via mitochondrial pathway and also causes a rise in the level of Bax and a decrease in the level of Bcl-2 in AGS and KATO III cells. OipA did not have any effect on cleavage of caspase 8 and FasL up-regulation, which are important in extrinsic apoptosis pathway (Fig. 4). There are reports in the literature indicating that, similar to OipA, VacA also induces apoptosis via mitochondrial pathway and modulates the Bax/Bcl-2 ratio in gastric epithelial cells^28^.

Yamaoka *et al*. have reported that the majority of *H*. *pylori*that were isolated from duodenal ulcer, gastric cancer and gastritis had functional OipA, suggesting that *H*. *pylori* with OipA functional status “On” cause more severe clinical outcomes^11^. Our results confirm this hypothesis by revealing a dose and time-dependent toxic effect for OipA towards cultured gastric epithelial cell lines. Furthermore, we confirmed the critical role of OipA in attachment of *H*. *pylori* to gastric epithelial cell lines (Fig. 1). Bacterial binding is the first step of bacterial colonization and *H*. *pylori* with a functional OipA attaches to gastric epithelial cells stronger than the bacteria either without OipA or with a none functional OipA^29^.

It is noteworthy that the effective OipA concentration on AGS and KTO-III were 0.256 and 0.5 µg/mL, respectively after 24 hrs, while the effective doses on both gastric cell lines are decreased to 0.128 and 0.256 µg/mL after 48 and 72 hrs of incubation. These results show that even lower dose of OipA can produce toxic effect when they are allowed to accumulate inside the cell for a longer time period.Our results also show that AGS cells were more vulnerable than KATO-III to OipA treatment (Figs 2 and 3) and OipA attached to AGS more than KATO III (Fig. 1). This could be explained by the fact that binding is critical for inducing cell death in host cells and AGS has more receptor for OipA protein.

It is also important to note that the Bax/Bcl-2 ratio for the cultured cell lines is inherently lower than in normal cells (Fig. 4), which means OipA protein can impact the Bax/Bcl-2 ratio in normal gastric epithelial cells more strongly than AGS and KATO III cell lines. Hence, the protein could potentially have a more severe apoptotic effects on normal gastric epithelial cells^30^. We, however, recommend that the effect of OipA on different signalling pathways be investigated in more detail in future investigations.

In summary, our data provides clear evidence regarding the strong pathogenesis of OipA. *H*. *pylori*is a class I carcinogen and one of the major causal agent of gastric cancer. It is critical that we gain solid understanding of the mechanism of action of this bacterium. It is important to note that *H*. *pylori* has several virulence factors. Numerous reports exist about several of these factors, with CagA and VacA being far more popular than others, skewing the literature and biasing our understanding of the disease. Our primary aim was to address this bias by focusing on the role of lesser-studied proteins like OipA in *H*. *pylori* pathogenesis. Without doubt, other virulence factors will also contribute to pathogenesis and a careful screening of all such proteins in mandated to find out the most effective (or a group of) proteins.

## Methods

### Ethics statement

Animal experiment was performed in accordance to regulations from the Animal Ethics Committee of Tarbiat Modares University, Faculty of Medical Sciences and the United States NIH guidelines (publication no. 85-23). The animal utilization protocols were approved by the Ethics Committee of Tarbiat Modares University, Faculty of Medical Sciences and all methods were performed in accordance with the relevant guidelines and regulations.

### OipA Production

The OipA protein was purified using the method previously described in the literature by the authors^31^. Briefly, the *oipA* gene was amplified from *H*. *pylori* strain 26695 by Polymerase Chain Reaction (PCR)^31^. PCR products were inserted into pJET1.2 vector (Novagen, USA), a suicide vector with blunt ends. For the isolation of *oipA* fragment with cohesive ends, the recombinant plasmid was subjected to a double digest protocol with *NcoI* and *XhoI* (Takara, Otsu, Japan). The isolated *oipA* fragment was inserted into a pET28a ( + ) vector (Novagen, USA). *OipA/pET28a* ( + ) was transferred into *E*. *coli* BL21 (DE3) (Fermentas, Lithonia) to produce OipA protein. Production of OipA was inducted in 2xYT medium (Merck, Germany) by 1 mM IPTG and the protein was purified by Ni–NTA affinity chromatography (Qiagen, USA). Polymyxin B sulfate (20 µg/mL) was added to purify OipA to remove lipopolysaccharide from the solution and the level of endotoxin was measured by Limulus amebocyte lysate assay kit (GenScrip).

### Rabbit polyclonal antibody production

A female New Zealand White rabbit (14 weeks) was used for rabbit polyclonal antibody production. For primary injection, the rabbit was immunized with 0.5 mg of OipA and complete freund adjuvant at 10 subcutaneous sits. For first, second and third boosters, the rabbit was injected with 0.25 mg of the protein and incomplete freund adjuvant on days 14, 28 and 42 at 4 subcutaneous sites. A 96-well ELISA plate was coated with 40 µg/mL OipA. The protein was incubated one hour at 37 °C followed by overnight incubation at 4 °C and ELISA test was performed as previously described in the literature^32^.

### Attachment of OipA to Human gastric cell lines

Human gastric cell lines (AGS and KATO-III) were obtained from the cell bank of the Pasteur Institute (Iran). AGS and KATO-III were cultured in RPMI 1640 supplemented with 100 µg/mL penicillin-streptomycin (Invitrogen, USA) and 10% fetal bovine serum (FBS). The cells were incubated at 37 °C and 5% CO2. RPMI 1640 and FBS were obtained from Invitrogen. The cells were seeded (5 × 10^4^ cells/well) in 96 well polystyrene flat-bottom micro titer plates and incubated for 48 hrs to make a confluent monolayer. The plates with and without cells were treated with various concentrations of OipA (2.5, 5, 10, 20 and 40 µg/mL) for 1 hr, after which wells were washed five times by phosphate buffered saline (PBS) and subsequently fixed with 2% formaldehyde. Heat-inactivated OipA and autologous rabbit serum were used as negative controls. Inactivation of OipA was performed with boiling 40 µg/mL of the purified protein for 30 minutes. OipA and primary antibody were added into wells without AGS and KATO-III to measure the level of non-specific binding of OipA and primary antibody to polystyrene plates.

Binding assay was performed by ELISA as described previously^32^. The OipA specific antibody (1:200) and HRP-conjugated anti-rabbit antibody (Sigma-Aldrich) (1:1000) were used as primary and secondary antibodies, respectively, for the ELISA assay. Heat-inactivated primary antibody was also used for control of antibody and Inactivation was performed with boiling of the rabbit serum for 30 mins.

### Viability Assay

Viability of OipA treated AGS and KATO-III was quantified using the MTT assay as previously described^33^. Briefly, 5 mg of MTT powder (Thiazolyl Blue Tetrazolium Bromide, Sigma-Aldrich) were dissolved in 1 mL PBS, pH 7.4 and filter-sterilized.The cells were cultured in 96-well polystyrene flat bottom micro titer plates (5 × 10^4^ cells/well) for 48 hrs. The cells were then treated with purified OipA at various concentrations (1ng/mL-100 µg/mL) or 100 µg/mL heat-inactivated OipA or 100 µg/mL BSA as negative controls for different co-incubation times (24, 48, and 72 hrs). Inactivation was performed with boiling of 100 µg/mL of the purified protein for 30 mins. After co-incubation, 20 µL of MTT solution were aseptically added to the wells and the plates incubated for 4 hrs (37 °C and 5% CO_2_), after which the supernatants were removed and 100 µL dimethyl sulfoxide (DMSO, Sigma-Aldrich) as a solubilisation solution were added to each well to dissolve the formazan crystals. After 10 mins of gentle shaking at room temperature, the optical density of each well was recorded at 570 nm.

### Annexin V/PI Apoptosis Assay

Apoptosis in AGS and KATO-III were detected by FITC-Annexin V/Propidium Iodide (BD Biosciences, USA) staining. The cells were seeded (2 × 10^5^ cells/well) in 24-well plates and incubated (37 °C and 5% CO_2_) for 48 hrs to make a confluent monolayer. The confluent cells were treated with different concentrations of OipA (2.5, 5, 10, 20, and 40 µg/mL) for 24 hrs. Heat-inactivated OipA (40 µg/mL) and BSA (40 µg/mL) were used as negative controls and inactivation was performed with boiling of the purified protein for 30 mins. The cells were subsequently trypsinized, harvested and washed three times with ice cold PBS. Double staining was performed by FITC-Annexin V and Propidium Iodide. The cells were re-suspended in 500 μL of 1X Binding Buffer, stained with 5 μL of Annexin V-FITC and 5 μL of propidium iodide, and incubated at room temperature for 5 min in the dark. Double stained cells were assessed by FACS scan System (BD Bioscience).

### Intracellular Signalling

Detection of caspase 3, 8, FasL, Bax and Bcl-2 proteins were carried out by immune blotting. Specific antibodies for caspase 3 (Biovision, Cat# 3015-100 “cleaved caspase-3”), FasL (Biovision,Cat# 3330-100), caspase 8 (Biovision, Cat# 3020-100 “procaspase-8), Bax (Biovision, Cat# 3032-100) and Bcl-2 (Alexis Biochemicals, Cat# ADI-AAS-070) were used for assessing the proteins expression. AGS and KATO-III were seeded (6 × 10^5^ cells/well) in 6-well plates and incubated for 48 hrs to make a confluent monolayer. The confluent cells were treated with OipA (20–40 µg/mL) or 40 µg/mL heat-inactivated OipA or 40 µg/mL BSA as negative controls for 24 hrs. OipA inactivation was performed with boiling of 40 µg/mL of the purified protein for 30 mins. The treated cells were washed and lysed by 50 µL lysis buffer containing protease and phosphatase inhibitor cocktails (1% Triton X-100, 50 mM Tris, pH 7.4, 150 mM NaCl containing 1 mM leupeptin, 1 mM pepstatin, and 100 mM phenyl methyl sulfonyl fluoride). The cell lysates were centrifuged for 10 mins at 400 × g and collected their supernatants. Total protein was assessed by Bradford assay and then 35 µg of protein was run on a 12% SDS-PAGE (Bio-Rad), followed by protein blotting on nitrocellulose and PVDF membranes as previously described in the literature^34^. Filters were blocked by non-fat dry milk in PBS containing 0.25% Tween-20 and probed with specific antibodies for caspase 3, 8, FasL, β-actin, Bax and Bcl-2. Protein bands were visualized by using a chromogenic detection method with a peroxidase conjugated secondary antibody and diaminobenzidine (Sigma-Aldrich). Scanning densitometer (Multi Gauge, V.3.0) was used for quantification of caspase 3, 8, FasL, β-actin, Bax and Bcl-2. The data was normalized with β-actin.

### Statistical Analysis

Data were analyzed using one-way ANOVA followed by Dunnett’s test (Origin Pro, v. 8.5.1., MA, USA) and *p* < 0.05 was chosen to indicate significant difference between data sets.

## Electronic supplementary material

Supplementary Information
