# Supplementary material for: Binding of the Helicobacter pylori OipA causes apoptosis of host cells via modulation of Bax/Bcl-2 levels
Source: Sci Rep. 2017 Aug 14;7:8036. doi: 10.1038/s41598-017-08176-7 (PMC5556102; doi:10.1038/s41598-017-08176-7)
Supplement: Supplementary file 1 — Supplementary Information [file 41598_2017_8176_MOESM1_ESM.pdf]

Binding of the *Helicobacter pylori* OipA causes apoptosis of host cells via modulation of Bax/Bcl-2 levels

Omid Teymournejad[1], Ashraf Mohabati Mobarez[1*], Zuhair Mohammad Hassan[2], Amin Talebi Bezminabadi[1]

[1]Department of Bacteriology, Faculty of Medical Sciences, Tarbiat Modares University, Tehran, Iran.

[2]Department of Immunology, Faculty of Medical Sciences, Tarbiat Modares University, Tehran, Iran.

***Author for correspondence:** Ashraf Mohabati Mobarez, Department of Bacteriology, Faculty of Medical Sciences, Tarbiat Modares University, Tehran/ Iran. Email: mmmobarez@modares.ac.ir , Tell and Fax: (+98 21) 82883862

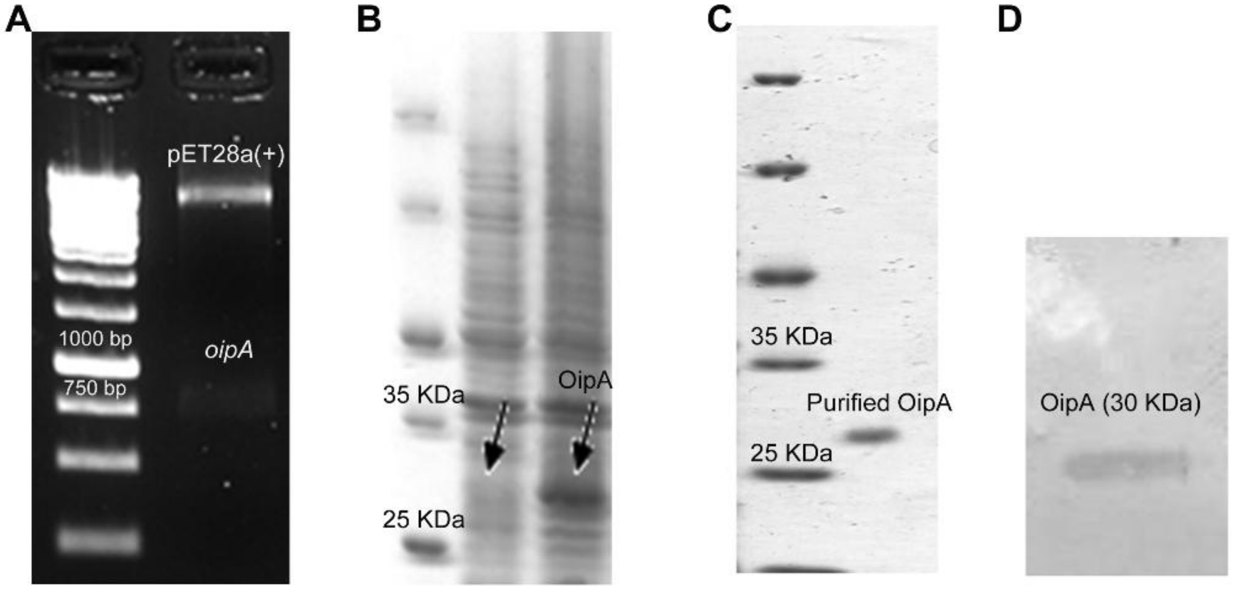

Fig. S1: Cloning, expression and purification of OipA.

Cloning of OipA confirmed with double digestion by *NcoI* and *XhoI* restriction enzymes (A). Coomassie blue stained 12.5% SDS-PAGE gel showing induction and purification of the expressed OipA protein (B and C). Western blot analysis using the OipA antibody showed a single 30-kDa protein on nitrocellulose paper (D).

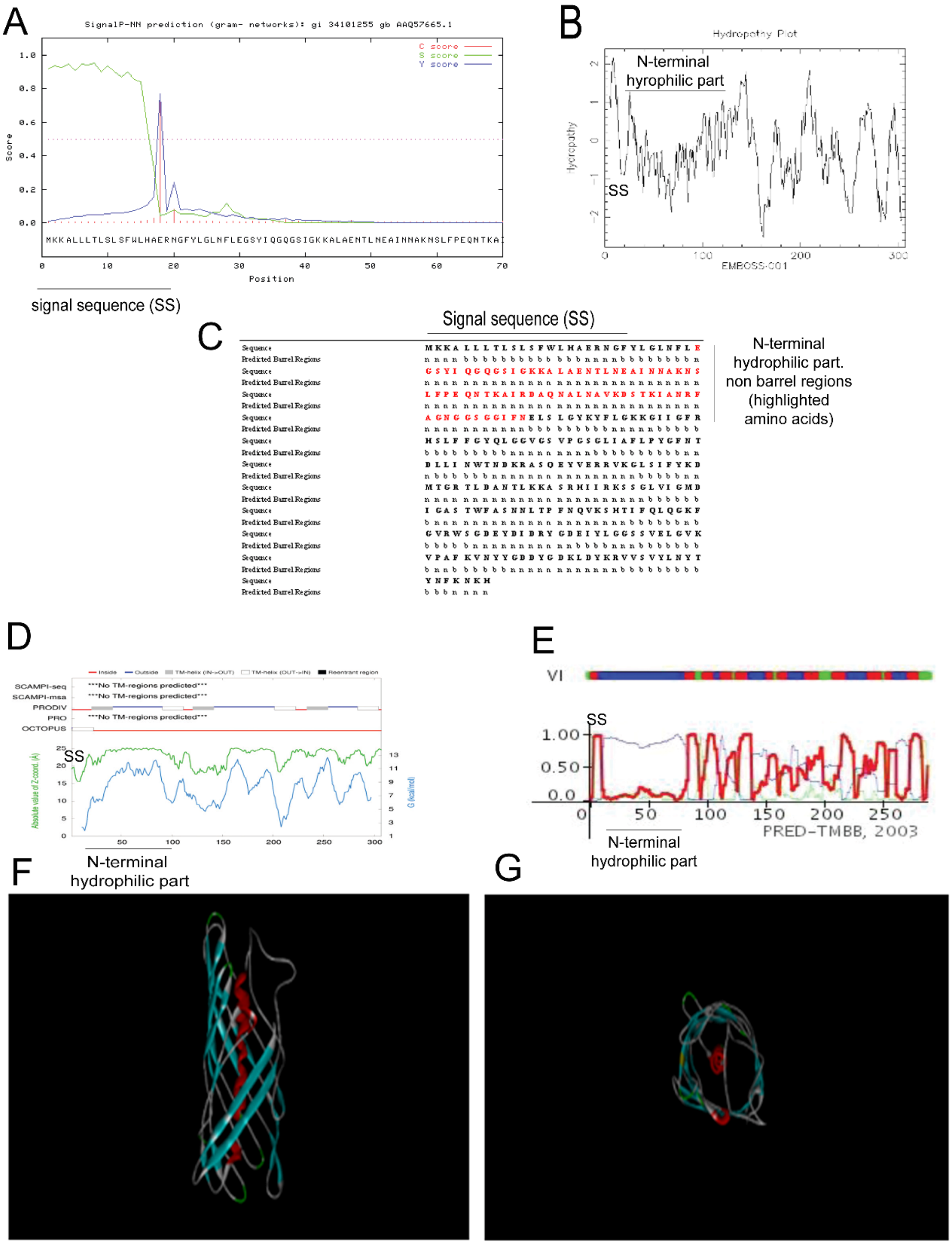
A
SignalP-NN prediction (gram- networks): gi 34101255 gb AAQ57665.1
C score
S score
Y score
Score
Position
MKKALLLTLSLSFWLHAERNGFYLGLNFLEGSYIQGQGSIGKKALAENTLNEAINNAKNSLFPEQNTKA
signal sequence (SS)
B
Hydropathy Plot
N-terminal hyrophilic part
SS
Hycropathy
EMBOSS:001
C
Signal sequence (SS)
Sequence M K K A L L L T L S L S F W L H A E R N G F Y L G L N F L E
Predited Barrel Regions n n n n b b b b b b b b b b b b b n n n n b b b b b b b b n
Sequence G S Y I Q G Q G S I G K K A L A E N T L N E A I N N A K N S
Predited Barrel Regions n n n n n n n n n n n n n n n n n n n n n n n n n n n n n n
Sequence L F P E Q N T K A I R D A Q N A L N A V K D S T K I A N R F
Predited Barrel Regions n n n n n n n n n n n n n n n n n n n n n n n n n n n n n n
Sequence A G N G G S G G I F N E L S L G Y K Y F L G K K G I I G F R
Predited Barrel Regions n n n n n n n n n n n b b b b b b b b b n n n n n b b b b b
Sequence H S L F F G Y Q L G G V G S V P G S G L I A F L P Y G F N T
Predited Barrel Regions b b b b b b b b b n n n n n n n n n b b b b b b b b b b n n
Sequence D L L I N W T N D K R A S Q E Y V E R R V K G L S I F Y K D
Predited Barrel Regions n b b b b b b n n n n n n n n n n n n n n n n b b b b b n n
Sequence M T G R T L D A N T L K K A S R H I I R K S S G L V I G M D
Predited Barrel Regions n n n n n n n n n n n n n n n n n n n n n n n n b b b b b b
Sequence I G A S T W F A S N N L T P F N Q V K S H T I F Q L Q G K F
Predited Barrel Regions b n n n n n n n n n n n n n n n n n n n n n b b b b b b n b
Sequence G V R W S G D E Y D I D R Y G D E I Y L G G S S V E L G V K
Predited Barrel Regions b b b b b n n n n n n n n n n n b b b b n n n b b b b b b b
Sequence V P A F K V N Y Y G D D Y G D K L D Y K R V V S V Y L N Y T
Predited Barrel Regions b n n b b b b b b n n n n n n n n n n n n b b b b b b b b b
Sequence Y N F K N K H
Predited Barrel Regions b b b n n n n
N-terminal hydrophilic part. non barrel regions (highlighted amino acids)
D
Inside
Outside
TM-helix (IN->OUT)
TM-helix (OUT->IN)
Reentrant region
SCAMPI-seq
SCAMPI-msa
PRODIV
PRO
OCTOPUS
No TM-regions predicted
SS
Absolute value of Z-coord. (Å)
G (kcal/mol)
N-terminal hydrophilic part
E
VI
SS
PRED-TMBB, 2003
N-terminal hydrophilic part
F
G

Fig. S2: *in-silico* prediction study on OipA showed that OipA is an auto-transporter.

Prediction of signal sequence of OipA using SignalP3.0 server (A). Hydropathy plot of OipA by EMBOSS Pepwindow server (B). Prediction of beta barrel of OipA in *H. pylori* outer membrane by TBBpred server; "n" refers to residues in non-barrel regions and "b" refers to residues in barrel region (C). Prediction of external and internal loops sequence of OipA by PRODIV-TMHMM server (D). Prediction of external and internal loops sequence of OipA by TMRPres2D server (E). Tertiary structure prediction of OipA by CPH model server (a protein homology server) and visualization with Discovery Studio Visualizer (F, G). The pattern recognition is according to profile-profile alignment guided by secondary structure and exposure predictions. The server has deleted approximately 30 hydrophilic amino acids from N-terminal sequence for finding the best match. Thus, the structures depicted in (F) and (G) are truncated forms of OipA and there must be thirty amino acid outside of the pore in N-terminal part.

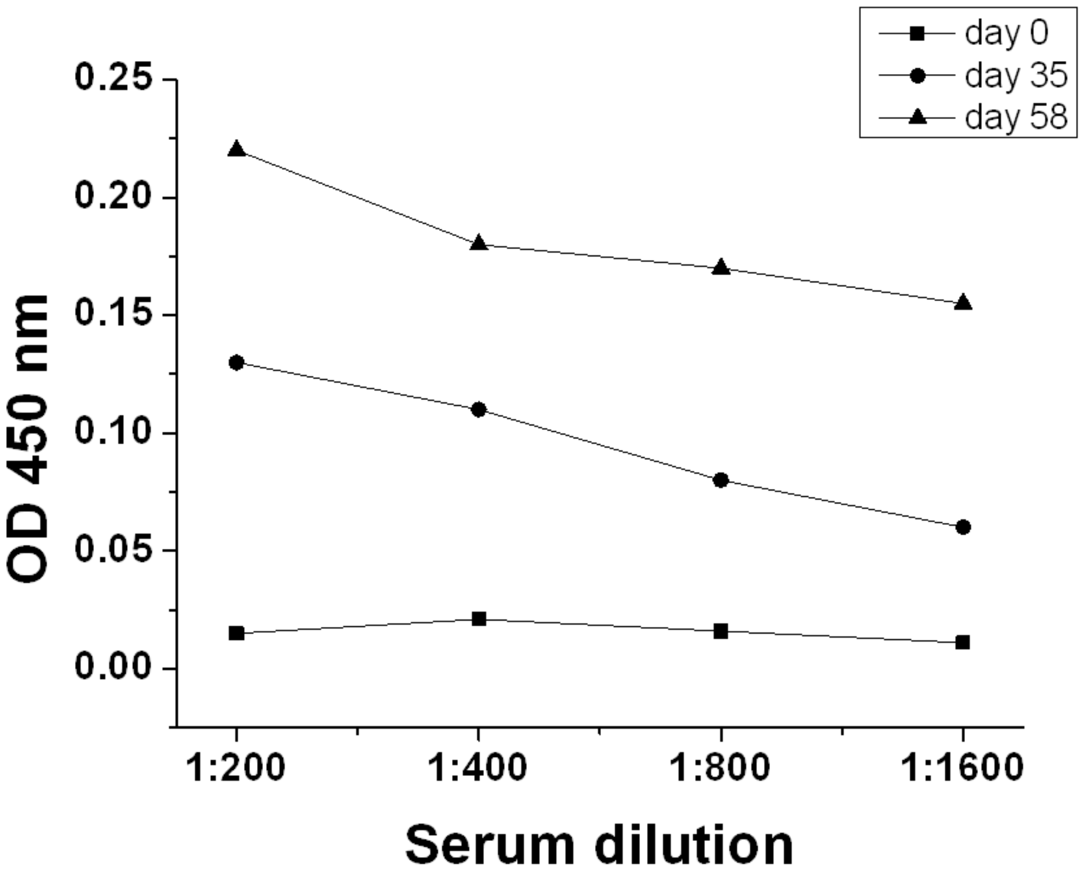

Fig. S3: Kinetic assessment of polyclonal antibody production against OipA.

A white female New Zealand rabbit was immunized 4 times with OipA with complete or incomplete frond adjuvant. The reactivity of diluted sera (serial dilutions from 1:200 to 1:1600) from the rabbit was measured at different time intervals by ELISA
